# Polymer-Peptide Modified Gold Nanorods to Improve Cell Conjugation and Cell Labelling for Stem Cells Photoacoustic Imaging

**DOI:** 10.3390/diagnostics11071196

**Published:** 2021-06-30

**Authors:** Dina Salah, Farahat S. Moghanm, Muhammad Arshad, Abdulaziz A. Alanazi, Salman Latif, Maie I. El-Gammal, Elmahdy M. Shimaa, Salah Elsayed

**Affiliations:** 1 Biophysics Group, Physics Department, Ain Shams University, Cairo 11566, Egypt; 2 Soil and Water Department, Faculty of Agriculture, Kafrelsheikh University, Kafr El-Sheikh 33516, Egypt; fsaadr@yahoo.ca (F.S.M.); shimaaelmahdy@agr.kfs.edu.eg (E.M.S.); 3 Department of Chemical Engineering, College of Engineering, King Khalid University, P.O. Box 394, Abha 61321, Saudi Arabia; moakhan@kku.edu.sa; 4 Department of Chemistry, College of Science and Humanities in Al-Kharj, Prince Sattam bin Abdulaziz University, Al-Kharj 11942, Saudi Arabia; Abdulaziz.alanazi@psau.edu.sa; 5 Department of Chemistry, College of Science, University of Hail, P.O. Box 2440, Hail 81451, Saudi Arabia; sl.alanazi@uoh.edu.sa; 6 Environmental Science Department, Faculty of Science, Damietta University, Damietta 35511, Egypt; mgammal_147@yahoo.com; 7 Agricultural Engineering, Evaluation of Natural Resources Department, Environmental Studies and Research Institute, University of Sadat City, Minufiya 32897, Egypt; salah.emam@esri.usc.edu.eg

**Keywords:** gold nanorods, photoacoustic imaging, stem cells, endocytosis

## Abstract

The use of gold nanorods (GNRs) as a contrast agent in bioimaging and cell tracking has numerous advantages, primarily due to the unique optical properties of gold nanorods which allow for the use of infrared regions when imaging. Owing to their unique geometry, Au NRs exhibit surface plasmon modes in the near-infrared wavelength range, which is ideal for carrying out optical measurements in biological fluids and tissue. Gold nanorod functionalization is essential, since the Cetyltrimethyl ammonium bromide CTAB gold nanorods are toxic, and for further in vitro and in vivo experiments the nanorods should be functionalized to become optically stable and biocompatible. In the present study, gold nanorods with an longitudinal surface plasmon resonance (LSPR) position around 800 nm were synthesized in order to be used for photoacoustic imaging applications for stem cell tracking. The gold nanorods were functionalized using both thiolated poly (ethylene glycol) (PEG) to stabilize the gold nanorods surface and a CALNN–TAT peptide sequence. Both ligands were attached to the gold nanorods through an Au–sulfur bond. CALNN–TAT is known as a cell penetrating peptide which ensures endocytosis of the gold nanorods inside the mesenchymal stem cells of mice (MSCD1). Surface modifications of gold nanorods were achieved using optical spectroscopy (UV–VIS), electron microscopy (TEM), zeta-potential, and FTIR. Gold nanorods were incubated in MSCD1 in order to achieve a cellular uptake that was characterized by a transmission electron microscope (TEM). For photoacoustic imaging, Multi-Spectral Optoacoustic Tomography (MSOT) was used. The results demonstrated good cellular uptake for PEG–CALNN–TAT GNRs and the successful use of modified gold nanorods as both a contrast agent in photoacoustic imaging and as a novel tracking bioimaging technique.

## 1. Introduction

The basic principle of photoacoustic imagining (PAI) is the detection of the sonic waves generated by the thermo-elastic expansion of a heated sample after laser excitation [1]. The sonic wave is detected by a piezoelectric crystal which converts the mechanical signal to an electrical signal [2]. The electrical signal is then processed in order to produce the final image of the sample. Multi-Spectral Optoacoustic Tomography (MSOT) is an advanced imaging technique that detects the thermal expansion generated by a sample because of its excitation using light irradiation. The resulted photoacoustic wave (ultrasound wave) gives a high-resolution image [3].

PAI requires high Near Infrared optical absorption contrast agents. Thus, gold nanorods are good candidates for photoacoustic imaging.

The optical properties of metallic nanoparticles result from the fact that when electromagnetic radiation hits such particles, it induces coherent, resonant oscillations of the free electrons in the nanoparticles. These oscillations are known as surface plasmon resonance. Huang and El-Sayed [4] studied the unique surface plasmon resonance properties of different gold nanoparticles shapes and its effect on the radiative and non-radiative properties, such as absorbance and scattering, of the nanoparticles. They also discussed the importance of these properties on the biomedical application of gold nanoparticles in cancer imaging, diagnosis, and therapy [5].

Gold nanorods have anisotropic plasmon resonances related to electron oscillation along two different directions, the transverse surface plasmon resonance (TSPR) around 520 nm, which is due to electron oscillation along the short axis, and the longitudinal surface plasmon resonance (LSPR), which can be tuned from 600 to 1100 nm and is due to electron oscillation along the long axis. The LSPRs of gold nanorods make them superlative candidates for biomedical imaging and therapeutic applications since they act as light absorber above 600 nm, where the need for near-infrared light is necessary due to the larger penetration depth involved in biological applications. Hence, the light in the region between 650–900 nm can have a penetration depth of several centimeters (depending on the type of the tissue) [6].

The seed mediated growth method is the most popular method for gold nanorod synthesis and was first developed by Jana et al. [7] and modified by Nikoobakht and El-Sayed [8]. The standard gold nanorod synthesis results in a gold nanorod that has its longitudinal surface plasmon resonance position around 800 nm, a property that makes it the best candidate for biomedical applications.

The Cetyltrimethyl ammonium bromide(CTAB) is essential in the seed mediated synthesis of gold nanorods. The seed mediated synthesis results in a tightly packed CTAB bilayer. This capping agent represents a challenge for further modifications of the gold nanorod surface. The use of CTAB gold nanorods for biomedical application is very restricted for many reasons such as its low colloidal stability in different solutions, incompatibility with another solvents, instability in long term storage, and, most importantly, reported CTAB cytotoxicity [9,10,11].

Generally, the functionalization of gold nanorods is challenging because of their large surface-to-volume ratio compared to spherical nanoparticles, and the large side-to-tip area ratio [12]. Many studies report different bio-functionalization methods for gold nanorods such as the use of thiolated molecules (e.g., mercaptopropionic acid, mercaptohexanoic acid, and thiolated poly (ethylene glycol) (SH-PEG)), which can replace CTAB [13,14]. Thiolated PEGs’ gold nanorods show high stability in aqueous solutions and various types of organic solvents such as ethanol, methanol, acetone, acetonitrile, dimethylformamide, dimethyl sulfoxide, and phosphate buffered saline with good biocompatibility [15]. These molecules provide stability against aggregation and can be used as anchor points for the further immobilization of biological molecules, given that thiol moieties have a high affinity for binding to a gold surface to form an Au–sulfur bond.

Proteins can also bind to gold nanorods via electrostatic interaction due to the positive charges of the CTAB bilayer. However, the physical adsorption appears unstable at high ionic strengths. All of these challenges dramatically impact the functionality of gold nanorods in biomedical applications [16].

Endocytosis is a process in which the cells import different extracellular objects such as viruses, microorganisms, signaling molecules, and nutrients. [17] Endocytosis has various pathways (e.g., cathrin-dependent and independent receptor-mediated endocytosis (RME), pinocytosis, and phagocytosis) [13]. RME is considered to be the most effective mechanism for nanoparticle cellular uptake, wherein functionalized nanoparticles bind to specific cell membrane receptors and, thereby, induce endocytosis [18].

The pinocytosis pathway represents a non-specific process to internalize biological fluids into cells [19], and phagocytosis occurs when phagocytic cells such as macrophages, dendritic cells, and neutrophils internalize foreign materials with sizes larger than 0.5 µm [20]. Many factors affect the endocytosis process including different sizes, shapes, and surface chemistries [21,22]. Gold nanoparticles are taken by cells via different routes, but their surface chemistry can be determined by the chemical composition and properties of the gold nanoparticle’s surface ligand on its surface such as the surface charge (i.e., positive, negative, or neutral), which can affect the nanoparticles’ cellular uptake efficiency and the endocytosis pathway (as has been reported in many studies) [23,24,25,26,27]. Specifically, Nativo et al., studied the endocytosis of 16 nm spherical nanoparticles into Hela cells using a transmission electron microscope. They found that the surfaces of gold nanoparticles were modified with different ligands such as citrate, thiolated PEG, the CALNN cell penetrating peptide, and nuclear localization signal [28]

A nanoparticle’s shape is also important for its biological behavior. For example, it has been reported that bacteria with different shapes, such as rods, spiral, and ellipsoids, have different endocytosis patterns [28]. Similarly, nanorods exhibit the highest uptake in different types of cells compared to spherical nanoparticles (in addition to their aspect ratio, which affects cellular uptake) [29,30]. The size of nanoparticles is an important factor in cellular uptake. Taruttis et al. [31] studied PEGylated gold nanorods of different sizes in the range which can be used in biomedical applications such as photoacoustic imaging and photothermal therapy [32,33,34]. They showed that different sizes resulted in different cellular uptake routes. They also confirmed the biocompatibility of gold nanorods functionalized with PEG molecules [31].

Different surface cell penetrating peptides such as the HIV-Tat peptide (TATp; RKKRRQRRR) and antennapedia homeodomain peptide (Antp or “penetrating”; RQIKIWFQNRRMKWKK) were successfully used to deliver different cargo, such as nanoparticles, into living cells. Both Kersemans et al. [35] and Krpetic et al. [36] have studied modified spherical gold nanoparticles with TAT peptide, following their cellular uptake pathway in cervical cancer cells. Stem cells are the original parent cells which can differentiate and grow to form different types of cells in tissues and organs. They maintain and replace the cells in the areas where they are present such as in blood, bone marrow, skin, muscles, and different organs. Stem cell research has become a novel hotspot and has provided new opportunities for regenerative medicine [37].

The combination of nanotechnology and stem cell research can ultimately lead to new technological opportunities as well as new challenges (e.g., the structure and properties of nanomaterials on the proliferation and differentiation of stem cells) [38]. Currently, nanoparticles such as quantum dots, magnetic nanoparticles, and gold nanorods can be used for the imaging and tracing of stem cells [38,39,40,41,42,43,44,45,46,47]. Comenge et al., used silica coated gold nanorods, which had a long LSPR at 730 nm, for the photoacoustic tracking of mesenchymal stem cells in mice. Their results showed a good resolution imaging and a good stability of the incubated cells in term of cell viability and cell differentiation properties. They reported using a high signal to monitor very small numbers of mesenchymal stem cells (as low as 2 × 10^4^ cells) via MSOT [3]. Lai et al. [48] used silica coated gold nanorods, which were modified by a poly arginine cell penetrating peptide, to enter tumor tropic cells. They reported a facile, robust, controllable, reproducible, and modified method to functionalize the gold nanorods with organosilica shells of different thicknesses to overcome the standard methods used to deposit silica into gold nanorods’ surfaces. The modified gold nanorods showed a good stability, viability, and good cellular uptake on murine macrophages cell lines. They had used a chitosan film which contained the silica coated gold nanorods to acquire a photoacoustic imaging with a homemade system. Chen et al. [32] studied the optical properties, photothermal stability, and photoacoustic response of PEGylated and silica coated gold nanorods. Their results showed a better and more stable photoacoustic signal in the case of silica-coated gold nanorods. Wang et al. [33] used mesoporous silica coated gold nanorods to deliver the imaging agent or therapeutic drugs to liver cancer cells. These mesoporous silica gold nanorods showed a minor long surface plasmon resonance shift, a higher light absorbance, a higher loading capacity, and low cytotoxicity. They assure gold nanorods’ potential application in therapy, imaging, and drug delivery for cancer cells [33].

Additionally, Dhada et al. [34] studied gold nanoparticles as a contrast agent for photoacoustic stem cells tracking. In their study, they used gold nanorods coated with near infrared dye. The stem cells loaded with gold nanorods showed a good cell viability for more than ten days, which allowed a long-term cell tracking.

Nanomaterials were also used in gene delivery applications, and in some therapeutic applications [49,50]. Recently, modified neural stem cells were used as nanoparticle carriers for cancer treatment, taking advantage of the fact that stem cells can overcome the brain barrier and its inherent properties with respect to brain tumors [51].

In this study, we used gold nanorods as a contrast agent for bioimaging and cell tracking, as they can be used within the infrared region due to their unique optical properties. Their unique shape allows them to have transverse surface plasmon resonance (TSPR) due to electrons oscillations on its short axis and long surface plasmon resonance (LSPR) due to electron electrons oscillation on its long axis. We used gold nanorods that had an LSPR at 800 nm, and a thiolated PEG and thiolated cell peptide sequence as a novel surface coating for its surface.

**Figure 1:**
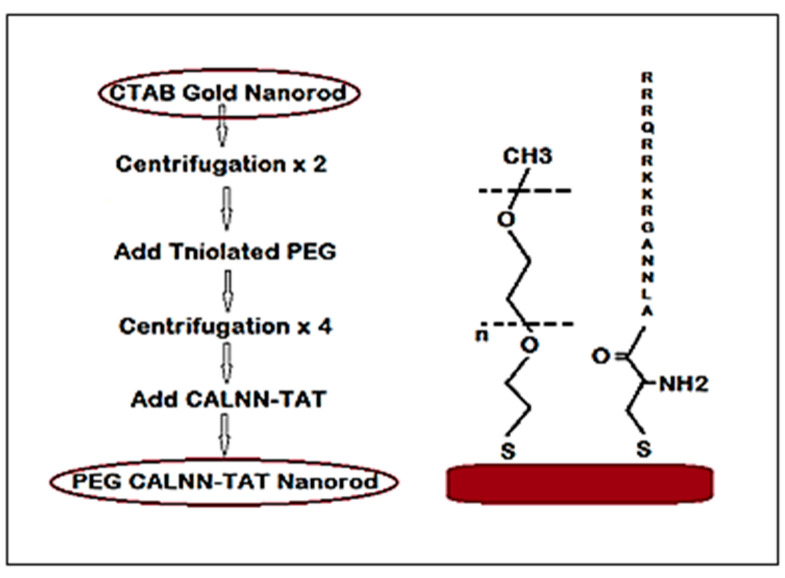
A schematic diagram of gold nanorod functionalization steps and the structure of the capping molecules utilized.

A schematic diagram of the modified gold nanorods is shown in Figure 1: Here, the thiolated polyethylene glycol and modified cell penetrating peptide follows the sequence CALNNAGRKKRRQRRR (i.e., the peptide sequence started with cysteine, which had a sulfur group).

The characterization and stability test for the synthesized gold nanorods showed good stability. This functionalization was considered to be direct and more biocompatible compared to the mostly commonly used and reported protocols for gold nanorod surface functionalization. Using the cell penetration peptide sequence ensures the endocytosis of gold nanorods into stem cells. The use of gold nanorods alone in diagnostic and therapeutic applications faces many problems that limit their clinical application such as their toxicity possibilities and their accumulation in the liver and spleen, as well as their kidney retention factors. Thus, using cells as cargo represents a good alternative to overcome these drawbacks.

In our experience, the major problems facing the cell tracking imaging using a contract agent are: (1) the fading of the contrast agent or its intensity attenuation and low resolution and (2) the short detection time. Hence, the use of gold nanorods in cell tracking and bioimaging have proved invaluable in their ability to overcome these problems.

Our results demonstrated that pegylated-CALNN–TAT gold nanorods showed a high cellular uptake by endocytosis into stem cells, as confirmed by TEM images, as well as a good stability and a high cell viability. It also gives a higher resolution MSOT signal compared to the control stem cells which were not incubated with gold nanorods.

The objective of this study was to evaluate the potential use of the modified gold nanorods without any further coating layers such as commonly used deposited silica layer or different nano-shells in bioimaging. Photoacoustic imaging represents a novel and noninvasive tracking imaging technique, and the use of modified gold nanorods enhanced the photoacoustic signal. Our thiolated PEG and cell penetrating peptide sequence showed good stability, cell viability and an enhanced high resolution photoacoustic signal using MSOT.

## 2. Materials and Methods

### 2.1. Gold Nanorods Synthesis

Gold nanorods were synthesized following Dickerson et al. [51] with slight modifications. As described, the seed solution was formed by adding 2.5 mL of 1 mM Gold (III) tetrachloroaurate trihydrate (HAuCl_4_) to 5 mL of 200 mM Cetyltrimethylammonium bromide (CTAB). This HAuCl_4_/CTAB mixture was kept at 27 °C, then 60 µL of ice-cooled 100 mM sodium borohydride (NaBH_4_) solution was slowly added to this mixture. This mixture was swirled before the seed solution was allowed to age for two hours. Then, 16 µL was added to the growth solution, consisting of 10 mL of 1 mM HAuCl_4_, 10 mL of 200 mM CTAB, 500 µL of 4 mM silver nitrate (AgNO_3_), and 140 µL of 78.8 mM ascorbic acid (A.A.).

UV/VIS spectra were obtained using a GENESYS 10S UV–VIS spectrometer. TEM samples of gold nanorods or seed NPs were prepared by placing 10 µL of aqueous sample dispersion onto TEM grids and allowing the solvent to naturally evaporate before the TEM images were obtained using a FEI TECNAI SPIRIT TEM (Hillsboro, OR, USA) at an operating voltage around 100 kV.

### 2.2. Gold Nanorods Functionalization

To functionalize the gold nanorods, a polymer coating and cell penetrating peptide of 100,000 thiolated PEG_5000_ molecules (polyethylene glycol monomethyl ether thiol) (HS-(CH_2_)_2_-O-(EG)_n_-CH_3_), with an average molecular weight 5000 g·mol^−1^, were added for each nanorod. The solution was left overnight, then centrifuged four times. 80% of the solution (i.e., the supernatant) was discarded and the remaining solution was re-suspended in milli-Q water for each centrifugation round. Thus, each centrifugation (rpm, 20 min) decreases the PEG concentration by a factor of around five. 3000 CALNN–TAT (Lifetein, Liverpool, UK), which is a modified cell penetrating peptide with sequence CALNNAGRKKRRQRRR was then added for each PEG GNR and left overnight to react without further centrifugation for the standard experiments.

For the gold nanorods’ functionalization characterization, first the hydrodynamic diameter of the nanorods under investigation was measured using a Zetasizer Nano ZSP DLS system equipped with a 532 nm laser, and an Avalanche photodiode detector (quantum efficiency >50% at 532 nm; Malvern Instruments Ltd.,Liverpool, UK). The same system was used for the zeta-potential measurement. Secondly, A BRUKER LUMOS FTIR Microscope was used for the gold nanorod characterization. Different samples were placed onto a Zinc Selenide window, and the solvent was completely evaporated before taking the FTIR spectra. For taking the FTIR spectrum, the window was placed in the sample holder stage, the microscope was focused on the sample area, and then the stage was moved to a sample-free area to get the background which was needed to calculate the sample spectrum. Finally, the stage was moved to different areas of the sample to take images and a spectrum for each area.

### 2.3. Cell Culture

Mesenchymal stem cells of mice (MSCD1) were cultured in high glucose Dulbecco’s Modified Eagle’s Medium (DMEM) (Catalog Number 21063045), supplemented with 10% fetal bovine serum (FBS). The cell culture dishes were kept at 37 °C in a humidified atmosphere of 5% CO_2_. Passages 10–25 were used for the experiments. A LEICA light microscope was used to image cells cultured in the cell dishes or wells.

### 2.4. Transmission Electron Microscopy (TEM) of Cells

The cells were cultured in 3.5 cm dishes, incubated with gold nanorods (or not as a control), and then fixed by the addition of 4% paraformaldehyde and 2.5% glutaraldehyde in a phosphate buffer (1 mL for each dish) for 1 h. Then, the cells were rinsed with a PBS buffer and post-fixed using a 1% aqueous solution of OsO_4_ (0.5 mL) for 1 h. Subsequently, the cells were washed with PBS and milli-Q H_2_O and stained with 0.5% uranyl acetate (0.5 mL, in 50% ethanol) for 1 h. Cells were then gradually dehydrated using a series of ethanol solutions (30, 60, 70, 80, 90 and 100%) and embedded in an epoxy resin. The resin was polymerized at 60 °C for 48 h. Ultrathin sections (70 nm) were cut using a diamond knife on a LEICA Ultramicrotome and mounted on Formvar coated copper grids. The sections were then post-stained with 5% uranyl acetate in 50% ethanol and 2% aqueous lead citrate solution and imaged with a FEI Tecnai Spirit TEM at around 100 kV using Analysis software (Soft Imaging Systems).

### 2.5. Photoacoustic Imaging

Multi-Spectral Optoacoustic Tomography (MSOT) was used for imaging stem cells loaded with PEG-CALNN–TAT gold nanorods against the control cells. For the MSOT experiments, MSCD1 was incubated with 0.5 nM PEG-CALNN–TAT modified gold nanorods dispersed in cell culture medium for a 24 h incubation time, as described above. After incubation, the excess of nanorods was removed and the cells were washed twice with 2 mL of a warmed PBS (1×) buffer, followed by a cell trypsinization step. The cell suspension was carefully collected and centrifuged (3000 rpm, 3 min), washed and re-suspended in PBS, and then cell counting was done by using a BIO-RAD TC 20 automated cell counter. Afterwards, 4% paraformaldehyde (PFA) was added to the cells and left for 20 min. Cells were then centrifuged one more time (3000 rpm, 3 min) and thereafter washed with PBS to remove the PFA excess. Then, they were kept in 500 µL Ficoll-PAQUE to be ready to be placed in the MSOT phantom, where GNR-labelled cells and control cells were placed in a phantom with two cavities. The MSOT was measured using excitation wavelengths from 680 to 900 (10 nm steps). A Mode linear algorithm was applied to reconstruct the image from ultrasound signals. Then, to identify gold nanorod-labelled cells, a linear regression algorithm, with the UV–VIS spectrum of the gold nanorods as a reference, was used [44].

## 3. Results and Discussion

### 3.1. Characterization of Functionalized Gold Nanorods

**Figure 2:**
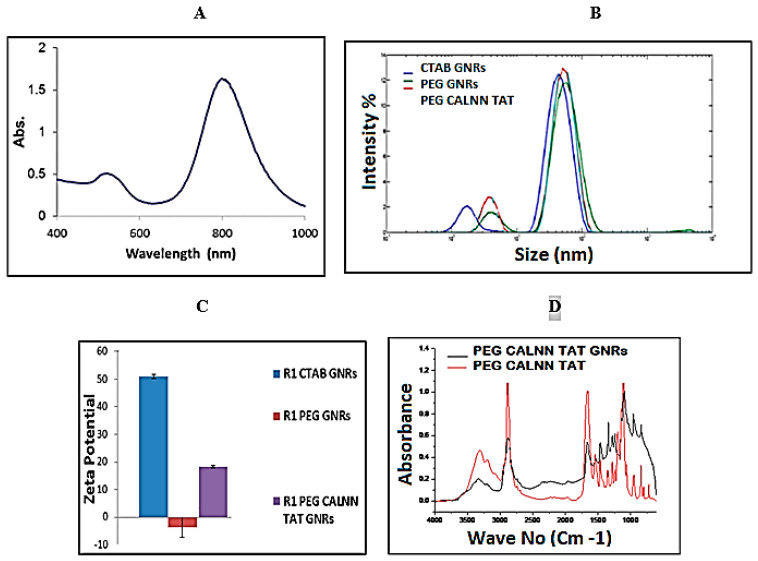
(**A**) UV–VIS spectrum of functionalized gold nanorods; (**B**) DLS size distributions of gold nanorods with different surface coating; (**C**) Zeta-potential changes during the functionalization steps of gold nanorods; and (**D**) FTIR spectrum show a sum of the PEG and CALNN–TAT spectra (red) and the PEG–CALNN–TAT gold nanorod spectrum (black) dried on a zinc selenide window.

The UV–VIS spectrum of the PEG–CALNN–TAT gold nanorods is shown in Figure 2A, which demonstrates a longitudinal surface plasmon peak around 800 nm. Surface modifications were detected by dynamic light scattering as shown in Figure 2B. After each functionalization step, the gold nanorods were placed in the DLS system as described in the experimental section. There is a change in the gold nanorod size, where the gold nanorods with PEG (MW 5000) on their surface have a larger size (green peak) than the gold nanorods with CTAB on their surface (blue peak), while the gold nanorods modified with PEG CALNN TAT (red peak) are in the middle between both CTAB and PEG.

Zeta-potential values for each gold nanorod surface modification are shown in Figure 2C. The results show a positive charge of CTAB gold nanorods, close to zero charge for PEG gold nanorods, and positive PEG–CALNN–TAT gold nanorods. Figure 2D indicates FTIR spectra of PEG–CALNN–TAT GNRs and a sum of the PEG and CALNN–TAT spectra. The results illustrate convincingly that both ligands are present on the gold nanorod surface as indicated by the perfect overlapping of both ligands characteristic peaks on the gold nanorod surface.

### 3.2. Microscopic Imaging

**Figure 3:**
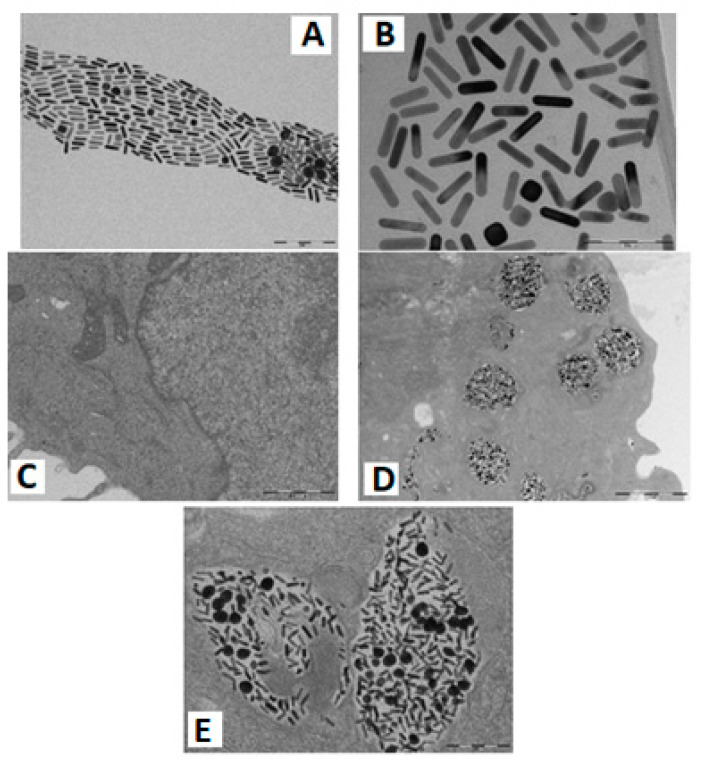
(**A**) TEM images for PEG–CALNN–TAT GNRs (scale bars are 200 nm) and (**B**) 100 nm; (**C**) stem cells control (without GNRs); (**D**) stem cells incubated with 0.5 nM PEG–CALNN–TAT GNR for 24 h scale bar 1000 nm; and (**E**) stem cells endosomes loaded with gold nanorods (scale bars are 200 nm).

TEM images of the functionalized gold nanorods (PEG–CALNN–TAT GNRs) and the stem cells displayed an endocytosis of 0.5 nM for the PEG–CALNN–TAT gold nanorods incubated for 24 h. A TEM image of a control cell (without GNRs) is shown in Figure 3.

### 3.3. Photoacustic Imaging (MSOT)

**Figure 4:**
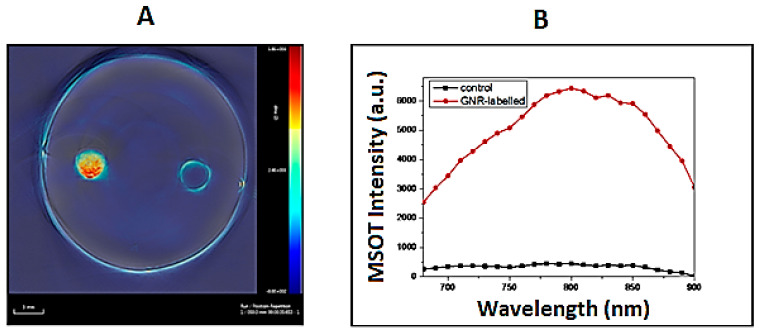
(**A**) MSOT signal intensity at 795 nm overlaid over a photo of the phantom. Left spot, cells loaded with gold nanorods; right spot, control cells; (**B**) MSOT integrated signal intensity at different wavelengths.

Photoacoustic imaging of the stem cells incubated with 0.5 nM gold nanorods for 24 h was done as described in experimental section. The MSOT results for two sets of experiments shown in Figure 4A represent the MSOT signal intensity overlaid over a photo of the phantom, where the left spot represents the cells that are incubated with gold nanorods, while the right spot is the control cells (without gold nanorods), and Figure 4B is the MSOT signal intensity at different wavelengths.

The results show a remarkable MSOT signal for cells incubated with gold nanorods, compared to control cells (without gold nanorods). Consequently, gold nanorods could work as an NIR contrast agent for photoacoustic imaging.

## 4. Conclusions

Gold nanorod surface modification is necessary because of the CTAB gold nanorods’ toxicity. In this study, the functionalization of gold nanorods was accomplished using a polymer and cell penetrating peptide (PEG–CALNN–TAT). Both bind to the gold surface through a thiol bond, as the gold surface has a good affinity to sulfur (which provides them with high levels of stability). Using gold nanorods alone in diagnostic and therapeutic applications faces many problems that limit their clinical application such as their toxicity possibilities and their accumulation in the liver and spleen, as well as their kidney retention factors. Thus, using cells as a cargo is a good alternative to overcome these drawbacks. Our modified gold nanorods demonstrated a good cellular uptake when they were incubated in stem cells for 24 h. Generally, the major problems facing cell tracking using a contract agent are the fading of the contrast agent, its intensity attenuation, and its low resolution and short detection time. Photoacoustic imaging results express the success of PEG–CALNN–TAT GNRs to work as a photoacoustic contrast agent which enhances the photoacoustic signal, its sensitivity, and its ability to overcome cell tracking problems. These results confirm the potential for using modified gold nanorods for different biomedical applications, especially as a novel tracking imaging technique in bioimaging and photoacoustic imaging.

## Data Availability

The data presented in this study are fully available in this article.
